# Serum Chemerin Levels in relation to Osteoporosis and Bone Mineral Density: A Case-Control Study

**DOI:** 10.1155/2015/786708

**Published:** 2015-06-15

**Authors:** Jing He, Ji-Chun Li, Hua Xie, Zhong-Hua Xu, Ya-Wen Sun, Qiao Shan

**Affiliations:** ^1^Department of Orthopedics, Jintan Hospital, Jiangsu University, Jintan 213200, China; ^2^College of Medicine, Zhejiang University, Hangzhou 310058, China

## Abstract

*Background*. To evaluate serum chemerin levels in patients with osteoporosis and healthy controls and to investigate the relationship between serum chemerin levels and bone mineral density (BMD). *Methods*. An age- and gender-matched case-control study was conducted. Pearson's correlation test was performed to investigate the relationship between serum chemerin levels and BMD. *Results*. There were 93 patients included in the osteoporosis group and 93 matched controls. Serum chemerin level was significantly higher in patients with osteoporosis (87.27±5.80 ng/mL) than patients in control (71.13±5.12 ng/mL) (*P* < 0.01). There was a negative correlation between femoral bone mineral density and chemerin in both groups (*R* = −0.395, *P* < 0.01 in osteoporosis group; *R* = −0.680, *P* < 0.01 in control) and also a negative correlation between lumbar bone mineral density with chemerin in both groups (*R* = −0.306, *P* < 0.01 in osteoporosis group; *R* = −0.362, *P* < 0.01 in control). *Conclusions*. Patients with osteoporosis presented a higher level of serum chemerin, which witnessed an inverse correlation with BMD. Further studies are needed to explore the role of chemerin in the pathophysiology of osteoporosis.

## 1. Introduction

Recent advances in metabolic research have led to the recognition that adipose tissue is an endocrine organ that secretes a variety of adipokines, which offer a connection between adipose tissue and the metabolic function of other organs, for example, bone [1]. Various adipokines have been proposed to be involved in bone metabolism via multiple effects on the formation and resorption of bone [2]. In contrast, bone, also as a source of bioactive factors, involves energy metabolism and then interacts with the anabolism and catabolism of adipose tissue [3].

The previous studies suggest that leptin, one of the remarkable adipokines, plays an essential role in osteogenesis and bone metabolism via multiple pathways and supports the possibility that leptin is involved in the onset or progression of various bone diseases, for example, osteoporosis [3, 4]. In the meantime, numerous studies have demonstrated altered levels of adiponectin and resistin in patients with osteoporosis [5–8].

Chemerin is a novel adipocyte-secreted factor, which plays an important role in adipogenesis, adipocyte differentiation, and insulin signaling [9, 10]. Previous studies have suggested that chemerin is related to obesity and metabolic abnormalities [11–13]. Moreover, chemerin also acts as a proinflammatory factor. The elevated secretion of proinflammatory cytokines induced by chemerin affects insulin sensitivity of adipocytes and distal tissues, such as liver and skeletal muscle [14]. Bones and skeletal muscles interacted with each other and are considered as one entity. As an adipose-derived signaling molecule, chemerin may play an essential role in the complex muscle-fat-bone axis. Therefore, we made a hypothesis that there was an association between serum chemerin levels and osteoporosis.

In the present study, we investigated the relationship between serum chemerin levels and bone mineral density (BMD) in patients with osteoporosis and healthy controls by conducting a case-control study.

## 2. Methods

### 2.1. Study Design and Patients

This observational study included 186 participants who were recruited from January 2014 to January 2015 at the outpatient clinic, the People's Hospital of Jintan. All participants had got BMD scans of their femoral neck and lumbar spines (L2–L4) by dual energy X-ray absorptiometry (Lunar Prodigy; General Electric Medical Systems, Milwaukee, WI, USA) on the purpose of physical examination [15–17]. A *T* score of ≤−2.5 was diagnosed as osteoporosis and ≥−0.5 was defined as normal [18]. 93 patients were enrolled because they met the criteria for osteoporosis diagnosis. The other 93 patients were selected to match on age and gender with those in the osteoporosis group (one-to-one paired). Subjects who suffered from malignant tumor, stroke, severe cardiopulmonary disease, virus hepatitis, liver cirrhosis, jaundice, renal insufficiency, endocrine diseases (Cushing's disease, acromegaly), and inflammatory rheumatism (rheumatoid arthritis, spondyloarthropathy) and those who smoked, abused alcohol (more than 20 g of ethanol per week), and used steroids or estrogens were excluded. All participants in this study provided written informed consent in official documents. This study conformed to the ethical guidelines of the 1975 Declaration of Helsinki as reflected in a priori approval by Ethics Committee of Jintan Affiliated Hospital, Jiangsu University.

Fasting blood samples were taken for the measurement of serum chemerin levels by using enzyme-linked immunosorbent assay (ELISA) (catalogue number SEA945Hu; Uscn Life Science Inc., China). In the meantime, routine blood chemistry analyses were performed at the laboratory department in our hospital. Data regarding age, sex, body mass index (BMI), having diabetes mellitus (DM) or hypertension, menstrual history, and serum concentrations of alkaline phosphatase (AKP), total cholesterol (TC), triglycerides (TG), low-density lipoprotein cholesterol (LDL-C), high-density lipoprotein cholesterol (HDL-C), fasting blood glucose (FBG), and insulin were recorded.

### 2.2. Statistical Analysis

Statistical analyses were performed using SPSS software (version 20.0). Continuous data were presented as mean ± standard deviation (SD), while categorical data were presented as absolute numbers. Statistical significance between the groups was evaluated using Student's *t*-test and* Chi*-squared test. Bivariate correlation analyses between BMD, serum chemerin levels, anthropometric data, and laboratory results were performed using Pearson's correlation test. In addition, partial correlation analyses were conducted to adjust for age and BMI. A *P* value of less than 0.01 (two-sided) was considered statistically significant.

## 3. Results

### 3.1. Patient Population

The basic characteristics of the 186 enrolled patients are presented in Table 1. There are 34 men and 59 women in each group. The mean age was 64 years. The mean BMI was 26.06 in osteoporosis group and 25.24 in control (*P* < 0.01). 15 patients with osteoporosis suffered from bone fracture while none had fracture in control. The comparison of DM status, hypertension status, number of postmenopausal women, and serum levels of TC, TG, HDL-C, FBG, and insulin between the two groups showed no statistical difference. However, mean LDL-C level was higher in control (*P* < 0.01), while mean AKP concentration was higher in osteoporosis group (*P* = 0.047). Serum chemerin level was significantly higher in patients with osteoporosis (87.27 ± 5.80 ng/mL) than patients in control (71.13 ± 5.12 ng/mL) (*P* < 0.01).

### 3.2. Correlation of Chemerin Level with Anthropometric and Biochemical Factors

Serum chemerin level was positively correlated with age (*R* = 0.395, *P* < 0.01 in osteoporosis group; *R* = 0.250, *P* = 0.016 in control), BMI (*R* = 0.391, *P* < 0.01 in osteoporosis group; *R* = 0.249, *P* = 0.016 in control), and FBG (*R* = 0.428, *P* < 0.01 in osteoporosis group; *R* = 0.340, *P* < 0.01 in control). In patients with osteoporosis, serum chemerin level was positively correlated with serum insulin level (*R* = 0.459, *P* < 0.01) and negatively correlated with LDL-C (*R* = −0.316, *P* < 0.01). In contrast, chemerin level positively correlated with LDL-C in nonosteoporosis subjects (*R* = 0.361, *P* < 0.01) (Table 2).

On the partial correlation analyses adjusted for age and BMI, plasmatic chemerin level was positively correlated with FBG (*R* = 0.342, *P* < 0.01) and insulin (*R* = 0.440, *P* < 0.01) in osteoporosis group and was positively correlated with LDL-C (*R* = 0.400, *P* < 0.01) and FBG (*R* = 0.300, *P* < 0.01) in control.

### 3.3. Correlation of BMD with Chemerin Level and Other Parameters

In osteoporosis subjects, femoral bone mineral density (FBMD) was negatively correlated with age (*R* = −0.293, *P* < 0.01), chemerin (*R* = −0.395, *P* < 0.01), FBG (*R* = −0.388, *P* < 0.01), and insulin (*R* = −0.388, *P* < 0.01). Similarly, lumbar bone mineral density (LBMD) was negatively correlated with age (*R* = −0.570, *P* < 0.01) and chemerin (*R* = −0.306, *P* < 0.01). Moreover, in control participants, FBMD was negatively correlated with chemerin (*R* = −0.680, *P* < 0.01) and LBMD negatively correlated with age (*R* = −0.510, *P* < 0.01) and chemerin (*R* = −0.362, *P* < 0.01). Nevertheless, neither FBG nor insulin had significant correlation with BMD in control (Table 3).

After adjusting for age and BMI in partial correlation analyses, there was a negative correlation between both FBMD (*R* = −0.689, *P* < 0.01) and LBMD (*R* = −0.318, *P* < 0.01) with chemerin in control, and a similar negative correlation between FBMD and chemerin (*R* = −0.340, *P* < 0.01) in osteoporosis group. However, no significant correlation was observed between LBMD with chemerin (*R* = −0.144, *P* = 0.172) in osteoporosis patients. In addition, FBMD was negatively correlated with FBG (*R* = −0.406, *P* < 0.01) and insulin (*R* = −0.410, *P* < 0.01) and LBMD negatively correlated with FBG (*R* = −0.272, *P* < 0.01) and insulin (*R* = −0.315, *P* < 0.01) in osteoporosis group. There was no significant correlation between BMD and FBG or insulin in control.

## 4. Discussion

This study has suggested that patients with osteoporosis present higher concentrations of serum chemerin than healthy controls. Furthermore, serum chemerin level has a negative correlation with FBMD and LBMD both in patients with osteoporosis and in healthy controls.

Chemerin is a novel adiponectin, which has been documented to be associated with metabolic syndrome. It is commonly accepted that incidence of osteoporosis is increased in patients with metabolic syndrome [19–23], which is characterized by obesity, dyslipidemia, insulin resistance, and nonalcoholic fatty liver disease [24, 25]. Because bone marrow mesenchymal stromal cell is the common precursor for osteoblasts and adipocytes, osteoporosis and metabolic syndrome might have common linkage [26]. Adipocytes secrete a spectrum of signaling molecules that impact bone homeostasis. These molecules include adiponectin, chemerin, resistin, leptin, and visfatin, which were reported to influence the function of osteoblasts and osteoclasts [27], and might be involved in the regulation of bone and mineral metabolism [28]. Previously, a number of human studies have suggested that adipokines, such as adiponectin, leptin, and resistin, might have a relationship with osteoporosis and BMD. In this case-control study, we aimed to examine the association between osteoporosis and plasmatic chemerin levels [5, 6, 8].

According to the results of this study, chemerin level was positively correlated with age, BMI, LDL-C, FBG, and fasting insulin, suggesting the relationship of chemerin with obesity, dyslipidemia, and insulin resistance. Furthermore, we have demonstrated that both FBMD and LBMD negatively correlated with chemerin level in osteoporosis patients and normal controls. After the adjustment of age and BMI, the correlation between LBMD and chemerin in patients with osteoporosis disappeared. In addition, in subjects with osteoporosis, partial correlation analyses suggested a negative correlation of BMD with FBG and insulin. It indicated an association among chemerin, insulin resistance, and bone metabolism, through the complex muscle-fat-bone axis.

To our knowledge, it is the first study to investigate whether serum chemerin level is altered in patients with osteoporosis and to evaluate the relationship between serum chemerin level and BMD. Strength of the present study was mainly the case-control design, matched for age and gender. In terms of limitations, it was not a population-based study. The possibility of selection bias had to be taken into consideration. And we did not measure plasmatic levels of other adipokines, such as adiponectin, leptin, and resistin, and did not evaluate the levels of bone metabolic markers, such as osteocalcin. Therefore, we failed to explore the relationship among these variables, BMD, and chemerin levels. Furthermore, this observational study precluded the establishment of a causal relationship between chemerin and osteoporosis. Further studies are needed to explore the causality and the underlying mechanism. Lastly, failure to include patients with osteopenia is also a limitation of our work. Due to being less aware of the bone health, subjects with osteopenia usually refuse to take full physical and biochemical examinations, which leads to limited number of eligible participants with osteopenia.

In conclusion, we reported a significant higher level of serum chemerin in patients with osteoporosis than normal controls. In addition, BMD presented an inverse correlation with serum chemerin levels. Further considerable studies are needed to demonstrate whether chemerin plays a role in the pathophysiology of osteoporosis and whether chemerin is qualified as a marker or predictor of osteoporosis.

## Figures and Tables

**Table 1:** Characteristics of the subjects.

	Osteoporosis	Control	*P* value
Number	93	93	
Male/female	34/59	34/59	
Age	64.60 ± 10.29	64.48 ± 10.29	0.443
BMI	26.06 ± 2.74	25.24 ± 2.10	<0.01
Number of patients with DM	34	28	0.437
Number of patients with hypertension	38	35	0.764
Number of postmenopausal women	55	50	0.239
Number of patients with bone fracture	15	0	<0.01
Chemerin (ng/ml)	87.27 ± 5.80	71.13 ±5.12	<0.01
FBMD (g/cm^2^)	0.63 ± 0.04	0.80 ± 0.07	<0.01
LBMD (g/cm^2^)	0.78 ± 0.04	1.04 ± 0.10	<0.01
TC (mmol/L)	6.35 ± 0.48	6.23 ± 0.45	0.077
TG (mmol/L)	1.69 ± 0.23	1.60 ± 0.36	0.067
HDL-C (mmol/L)	1.47 ± 0.29	1.50 ± 0.35	0.356
LDL-C (mmol/L)	3.00 ± 0.24	3.12 ± 0.24	<0.01
AKP (U/L)	99.29 ± 21.31	92.99 ± 21.03	0.047
FBG (mmol/L)	6.04 ± 1.20	5.81 ± 1.13	0.136
Insulin (mU/L)	17.13 ± 4.52	18.12 ± 3.82	0.079

Data are presented as mean (standard deviation).

BMI: body mass index; DM: diabetes mellitus; FBMD: femoral bone mineral density; LBMD: lumbar bone mineral density; TC: total cholesterol; TG: triglycerides; HDL-C: high-density lipoprotein cholesterol; LDL-C: low-density lipoprotein cholesterol; AKP: alkaline phosphatase; FBG: fasting blood glucose.

**Table 2:** Bivariate correlation analyses between serum chemerin levels with anthropometric and laboratory variables.

	Osteoporosis group				Control group			
	Unadjusted		Adjusted^*∗*^		Unadjusted		Adjusted^*∗*^	
	*R* value	*P* value	*R* value	*P* value	*R* value	*P* value	*R* value	*P* value
Age	0.395	<0.01	NA	NA	0.250	0.016	NA	NA
BMI	0.391	<0.01	NA	NA	0.249	0.016	NA	NA
TC	−0.204	0.049	−0.119	0.263	−0.140	0.182	−0.113	0.287
TG	0.090	0.393	0.176	0.094	0.189	0.070	0.053	0.618
HDL-C	0.259	0.012	0.135	0.201	0.128	0.220	0.129	0.224
LDL-C	−0.316	<0.01	−0.264	0.011	0.361	<0.01	0.400	<0.01
AKP	−0.049	0.643	−0.015	0.891	0.106	0.314	0.132	0.213
FBG	0.428	<0.01	0.342	<0.01	0.340	<0.01	0.300	<0.01
Insulin	0.459	<0.01	0.440	<0.01	0.085	0.420	0.051	0.633

BMI: body mass index; TC: total cholesterol; TG: triglycerides; HDL-C: high-density lipoprotein cholesterol; LDL-C: low-density lipoprotein cholesterol; AKP: alkaline phosphatase; FBG: fasting blood glucose; NA: not applicable.

^*∗*^Partial correlation analyses were performed to adjust age and BMI.

**Table 3:** Bivariate correlation analyses between bone mineral density with serum chemerin levels and anthropometric and laboratory variables.

	Osteoporosis group								Control group							
	FBMD				LBMD				FBMD				LBDM			
	Unadjusted		Adjusted^*∗*^		Unadjusted		Adjusted^*∗*^		Unadjusted		Adjusted^*∗*^		Unadjusted		Adjusted^*∗*^	
	*R* value	*P* value	*R* value	*P* value	*R* value	*P* value	*R* value	*P* value	*R* value	*P* value	*R* value	*P* value	*R* value	*P* value	*R* value	*P* value
Age	−0.293	<0.01	NA	NA	−0.570	<0.01	NA	NA	−0.219	0.035	NA	NA	−0.510	<0.01	NA	NA
BMI	−0.024	0.822	NA	NA	0.051	0.626	NA	NA	0.005	0.963	NA	NA	−0.020	0.848	NA	NA
Chemerin	−0.395	<0.01	−0.340	<0.01	−0.306	<0.01	−0.144	0.172	−0.680	<0.01	−0.689	<0.01	−0.362	<0.01	−0.318	<0.01
TC	0.108	0.303	0.013	0.901	0.117	0.262	−0.096	0.365	0.129	0.217	0.124	0.243	−0.043	0.685	−0.080	0.451
TG	−0.047	0.655	−0.201	0.056	0.106	0.310	−0.218	0.038	0.133	0.203	0.190	0.071	0.026	0.804	0.138	0.191
HDL-C	−0.215	0.039	−0.314	<0.01	0.022	0.833	−0.132	0.212	0.257	0.013	0.229	0.029	0.150	0.151	0.073	0.491
LDL-C	0.003	0.976	−0.071	0.504	0.126	0.229	−0.007	0.945	−0.144	0.169	−0.143	0.175	−0.036	0.733	−0.032	0.762
AKP	0.181	0.082	0.173	0.101	−0.143	0.171	−0.209	0.047	−0.199	0.056	−0.208	0.048	−0.166	0.111	−0.207	0.049
FBG	−0.388	<0.01	−0.406	<0.01	−0.225	0.030	−0.272	<0.01	−0.138	0.187	−0.187	0.075	−0.128	0.220	−0.229	0.029
Insulin	−0.388	<0.01	−0.410	<0.01	−0.243	0.019	−0.315	<0.01	−0.125	0.234	−0.097	0.359	−0.156	0.135	−0.100	0.346

^*∗*^Partial correlation analyses were performed to adjust age and BMI.

FBMD: femoral bone mineral density; LBMD: lumbar bone mineral density; BMI: body mass index; TC: total cholesterol; TG: triglycerides; HDL-C: high-density lipoprotein cholesterol; LDL-C: low-density lipoprotein cholesterol; AKP: alkaline phosphatase; FBG: fasting blood glucose; NA: not applicable.
